# Plasma Protein Biomarkers for the Cognitive Functions in Women Living with HIV

**DOI:** 10.1002/alz.083703

**Published:** 2025-01-09

**Authors:** Wei Li, Leah H Rubin, Yanxun H Xu, Ge Wang, Daniel Li, David E Vance

**Affiliations:** ^1^ University of Alabama at Birmingham, Birmingham, AL USA; ^2^ Johns Hopkins University, Bethesda, MD USA; ^3^ Johns Hopkins University, Baltimore, MD USA; ^4^ Tongji Hospital, Tongji Medical College, Huazhong University of Science and Technology, Wuhan China; ^5^ University of California at Los Angeles, Los Angeles, CA USA; ^6^ UAB, Birmingham, AL USA

## Abstract

**Background:**

Individual plasma protein biomarkers have been shown to correlate with cognitive performance in people with HIV. This study aimed to investigate the association between plasma proteomic signatures and cognition in virologically well‐controlled women with HIV.

**Method:**

77 women with HIV from three Women's Interagency Study (WIHS) sites completed neuropsychological testing and a blood draw. Targeted protein biomarkers were analyzed using a multiplexing method. Random forest analysis was used to identify the top 10 biomarkers that positively or negatively associated with domain‐specific or global neuropsychological function. Next, ingenuity pathway analysis was used to facilitate data interpretation.

**Result:**

Plasma protein biomarkers are either related to domain‐specific or overall cognition: tumor necrosis factor receptor 1 (TNF RI), TNF‐RII, interleukin 1 receptor 1 (IL‐1RI), and IL‐6R are negatively associated with attention/working memory; IL‐7 and CD40 are positively associated with executive function; IL‐1RI, hepatocyte growth factor (HGF), transforming growth factor beta‐1(TGFβ1), TGFβ2, and sonic hedgehog (SHH) are negatively associated but IL‐7 is positively associated with fluency; IL‐9, IL‐15, and IL‐21R are positively associated with motor function; Brain‐derived neurotrophic factor (BDNF), HGF, SHH, and vascular endothelial growth factor (VEGF) are negatively associated with processing speed. HGF, VEGF, and insulin are negatively associated with global neuropsychological function. Based on the ingenuity pathway analysis, one to three signaling networks were proposed for associating plasma biomarkers with each domain‐specific or global neuropsychological function.

**Conclusion:**

It is likely that a panel of plasma protein biomarkers can be used to predict domain‐specific or global neuropsychological function. Multiple signaling networks might explain cognitive functions in Women with HIV.

